# Supplementary material for: The interactome of intact mitochondria by cross-linking mass spectrometry provides evidence for coexisting respiratory supercomplexes
Source: Mol Cell Proteomics. 2017 Dec 8;17(2):216–32. doi: 10.1074/mcp.RA117.000470 (PMC5795388; doi:10.1074/mcp.RA117.000470)
Supplement: Supplemental Data [file supp_RA117.000470_133922_0_supp_23978_fzffwf.zip › spectra_annotation/mito_DR_spectra_annotation/33-1-1-1-6-1.pdf]

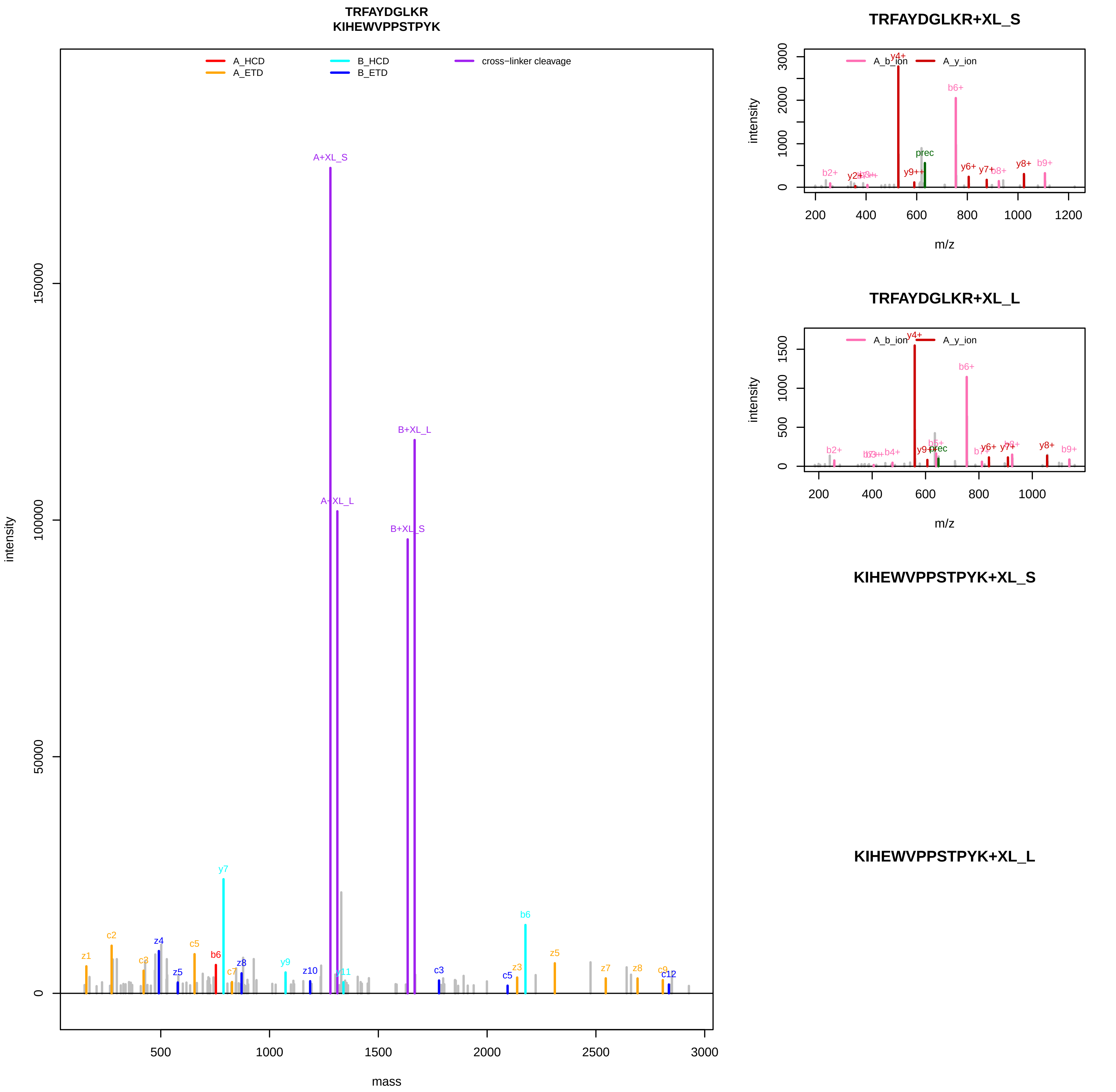
TRFAYDGLKR
KIHEWVPPSTPYK
A_HCD
A_ETD
B_HCD
B_ETD
cross−linker cleavage
A+XL_S
A+XL_L
B+XL_L
B+XL_S
y7
b6
z1
c2
c3
z4
z5
c5
b6
c7
z8
y9
z10
y11
c3
c5
z3
z5
z7
z8
c9
c12
intensity
mass
0
50000
100000
150000
500
1000
1500
2000
2500
3000
TRFAYDGLKR+XL_S
A_b_ion
A_y_ion
y4+
b6+
prec
b2+
y2+
y9++
y6+
y7+
b8+
y8+
b9+
intensity
m/z
TRFAYDGLKR+XL_L
A_b_ion
A_y_ion
y4+
b6+
b2+
b4+
b5+
y9+
prec
b7+
y6+
y7+
b8+
y8+
b9+
intensity
m/z
KIHEWVPPSTPYK+XL_S
KIHEWVPPSTPYK+XL_L
